# Some Thoughts Suggested by the Local or Medicinal Treatment of Cataract1Read at Section of Ophthalmology, Otology, Rhinology and Laryngology, Buffalo Academy of Medicine, April 16, 1900.

**Published:** 1900-07

**Authors:** B. H. Grove

**Affiliations:** Buffalo, N. Y.


### SOME THOUGHTS SUGGESTED BY THE LOCAL OR MEDICINAL TREATMENT OF CATARACT.[1]

By B. H. GROVE, M. D., Buffalo, N. Y.

All natural sciences have progressed more or less through empiricism. Many of the accepted theories of one age appear to that which follows the rankest nonsense. This applies to the history of medicine as truly as to that of any other science. I remember in my college days in reading the history of medicine, nothing seemed to me more nauseating than the many abominable messes that were recommended and extolled by the profession of ancient times, for relief of various pathological conditions. For example, one of the more refined prescriptions was a pill made out of the skull of an executed criminal for the bite of a mad dog. The revival of organotherapy is interesting and shows how cycles of medicine like history

1. Read at Section of Ophthalmology, Otology, Rhinology and Laryngology, Buffalo Academy of Medicine, April 16, 1900.

repeat themselves. The ancient Egyptians appear to have placed much reliance upon the viscera of animals and the excrement of the gazelle as powerful remedies for certain diseased conditions of the eye, especially conjunctivitis.

Other forms of eye inflammation were relieved by the use of the human brain. Some portions of it were to be mixed with honey and rubbed on the eye at night and other portions were to be pulverised and used upon the eye in the morning. Blood of various animals was supposed to be very effective and edema or chemosis of the conjunctiva was speedily relieved by the milk of a nursing woman. This last mentioned remedy has been recommended to my actual knowledge even by some members of our local profession. However it is unnecessary to go back to the ancients; the last twenty years have sufficed to demonstrate the rise and fall of various queer methods of treatment, for example, I might mention, hydrogen sulphide by rectal injections for the cure of tuberculosis. This always seemed to me the acme of ridiculousness, and still I know there are members of the Academy who have testified to the cure of several cases by this method of treatment. I suppose today among the rubbish of some of the drug stores one might find the apparatus formerly used for this application of sulphuretted hydrogen. Along these lines it is interesting to read, Currents and Counter Currents, by Oliver Wendell Holmes.

We are not surprised, of course, when we read the startling testimonials of the laity. Recently one of the managers of a mineral water concern among whose stockholders are several physicians of good repute, told me he had received a testimonial from one of his patrons saying he was cured of an inguinal hernia by indulging freely in this peculiar liquid. Now it does seem that some physicians themselves are not without blame in this respect, judging from the various certificates that come to you and me in nearly every day's mail. A favorite expression of the fadist is "no one is so blind as he who will not see." It is, of course, axiomatic that if no one experimented nothing would be discovered and I am aware many of the best things in medicine have been discovered simply by accident. Still this does not mean that conclusions should be hastily and falsely drawn. What is necessary is true scientific methods; first, accurate diagnosis and, secondly, a thorough observation and a proper regard for coincidences. For about one year I, and doubtless many of you, have received literature from a certain St. Louis concern extolling the virtues of succus cinerariæ maritimæ, for numerous eye affections, including the absorption of various forms of cataracts. This precious juice is dark in color and has a pungent taste and is doled out in vials, each holding about one drachm. The price is $1.00 per vial, or twelve for $9.00. At first I threw the circulars in the waste basket, but from time to time more were received. Finally, my curiosity was aroused, but my incredulity persisted. The circular announces, quoting from an exhaustive article in the *British Medical Journal,* how this juice has been employed with marvelous effects That it is used by eminent physicians and ophthalmologists at home and abroad. That while its primary action is directed to capsular and lenticular cataracts many claim excellent results in other diseases of the eye. In iritis it overcomes inflammations, removes adhesions and causes rapid absorption of all exudates, etc.

One physician reports as follows:

My patient is steadily improving from the use of cineraria. Her case is a peculiar one. Cataracts have been forming in both eyes for ten years, but the lens have never become entirely hardened. She is being so much benefited by this remedy that it will not be necessary to operate, although several prominent physicians have advised this as the only means of relief.

While I considered the claims a swindle, still I wished to satisfy my curiosity, so I procured some of the drug and used it for three or four months in six cases, explaining to my patients at the time my disbelief in its merits. I tested my patients carefully, and demonstrated to my satisfaction the therapeutic value of the drug. During this time some general practitioners had asked me about this juice, and I was consulted by some patients in the city and vicinity who had been treated by their family physicians with this cineraria maritima. Notwithstanding this experience these notes would not have been written had I not read the following in such a reputable publication as the *Medical Record*, under date of January 27, 1900:

Succus cineraria maritima is said to act in the absorption of cataract when dropped into the eye daily, two or three drops at a sitting. It is said that in many instances the results are "nothing short of miraculous."

It seemed that judging from my experience I should not allow this statement to pass unchallenged. Besides, my curiosity was aroused to learn if this drug was being employed, and if so to what degree by the profession generally. Accordingly, I sent out a few hundred cards and I learned that many physicians were interested in the subject and that not a few had made use of the drug. Many wanted to know my opinion and were just about to send for the so-called magic fluid, but would wait to learn of my experience.

I am obliged to say therefore that after a fair and impartial trial I consider this drug without any merit whatsoever and simply one of the many delusions pure and simple. This opinion is also corroborated by the members of the Buffalo Ophthalmological Society, at least by all of those who have investigated the subject and by other oculists in this vicinity. To be sure, some of the physicians in general practice have returned me answers apparently in favor of the drug, but I imagine their diagnosis and examinations were not carefully made and that their patients were not afflicted with cataract. I surmise this because recently I have been consulted by a few who were being treated by cineraria for cataract because the pupil did not seem clear and black. Thorough examination, however, did not disclose a cataractous eye. Often a slight conjunctivitis, acute or otherwise, will cause an impairment of the eye-sight. The vision improves it may be with or without treatment and thus coincidentally the drug being used will get the credit. Although up to the present time treatment for cataract otherwise than operative has not attained any results, we must not deny the possibility that the time will come when it will become possible to arrest further development of a beginning cataract, or even clear up an opacity which has already developed. The cataractous lens is not to be looked upon as the result of excessive changes necessarily due to age, but rather the result of nutritive disturbances, chemical in nature.

From the earliest times not only have the most manifold suggestions been offered to bring about by medicinal treatment the disappearance of a beginning cloudiness of the lens in cases of partial and even total cataracts, but frequently those have come forward who claim good results from such efforts. But I will spare you from enumerating all those things which have been tried and reported worthy of a trial, for this would necessitate my beginning with honey and saffron, cooked in the bile of hyenas, and mentioning all the remedies down to dilute phosphoric acid, which fifteen or twenty years ago was praised in even scientific papers both in France and Germany as a universal remedy for grey cataract. I must not forget to refer to the method pursued by Dr. Kalish, of New York, some few years ago. The disappearance of incipient opacities has been at various times claimed to be effected by massage of the globe. He reported several cases and claimed to have attained some success, but the evidence is not convincing and the success has been very partial.

Massage is however a two-edged weapon, because in many cases instead of retarding the growth of cataract it has hastened it. The claims of galvanism likewise have no reliable foundation. I trust you have not been too much wearied with these rambling notes. In looking up this subject of the use of cineraria, I have at least the satisfaction of knowing that I have served some of my friends and have satisfied myself of the worthlessness of this drug for eye affections.

334 Pearl Street.

